# Climate change adaptation and the back of the invisible hand

**DOI:** 10.1098/rstb.2022.0406

**Published:** 2023-11-06

**Authors:** H. Clark Barrett, Josh Armstrong

**Affiliations:** ^1^ Department of Anthropology, University of California, Los Angeles, CA, 90095-1553, USA; ^2^ Department of Philosophy, University of California, Los Angeles, CA, 90095-1553, USA

**Keywords:** cultural evolution, niche construction, invisible hand, Anthropocene, climate change, adaptation

## Abstract

A good deal of contemporary work in cultural evolutionary theory focuses on the adaptive significance of culture. In this paper, we make the case that scientifically accurate and politically feasible responses to the climate crisis require a complex understanding of human cultural practices of niche construction that moves beyond the adaptive significance of culture. We develop this thesis in two related ways. First, we argue that cumulative cultural practices of niche construction can generate stable equilibria and runaway selection processes that result in long-term existential risks within and across cultural groups. We dub this *the back of the invisible hand*. Second, we argue that the ability of cultural groups to innovate technological solutions to environmental problems is highly constrained in ways that are exacerbated by sustained intergroup conflict, inequality and by inherently unpredictable cascades in climate change and human migration patterns. After developing these theoretical points about human cultural practices of niche construction in detail, we conclude our discussion with some tentative practical suggestions about the way that cultural evolutionary history can more fruitfully be used in efforts to remit the climate crisis and contribute to sustainable practices of human climate change adaptation.

This article is part of the theme issue ‘Climate change adaptation needs a science of culture’.

## 1. Introduction

Climate change is one of the most profound sources of existential risk in our times. It threatens not just irreversible changes to the globe and to human lifeways, but also potentially an end to our species. In scientific and political spheres as well as the popular imagination, climate change has replaced nuclear war as perhaps the most feared threat to our continued persistence [1].

Entangled in discussions of climate change are debates about its causes, consequences and potential remedies. The concept of the Anthropocene, defined by some as the age of human-induced (and geologically visible) global change, has entered the popular imagination and scientific discourse [2]. There is widespread agreement that much or most global climate change is human-induced, the result of human activities on multiple scales, such as the emission of greenhouse gases as a byproduct of energy extraction, deforestation and other commercial activities [3]. The consequences of human-induced climate change are difficult to predict, but substantial scientific effort has been put towards predicting a point-of-no-return rate of global warming that the global community must work to avoid. Because the relationships between human activities and global warming are complex, substantial effort has also been put towards understanding the input–output relationships between these activities and the climate. This work, in turn, has informed discussions of how we might slow the rate of warming through various national or international policies, as well as other means of responding to or mitigating the harmful effects of climate change.

There is debate over the causes of climate change, with some arguing that it is not caused by humans at all [4]. However, the majority view of scientists is that climate change is at least in part caused by human activity [3]. Some have appealed to the biological concept of niche construction [5] as a useful theoretical framework for understanding the reciprocal relationships between human activity and climate change [6,7]. We agree that this is a useful perspective, and it is the one we adopt here.

For reasons that we will explain, the centrality of niche construction in our evolutionary history gives neither reason for optimism nor pessimism about the future of human welfare in the Anthropocene. It merely posits that humans and ‘the environment' are not separate realms or compartments, but mutually co-constituted. Thus, our activities do (partly) construct the environments in which we live, and *vice versa*—but this insight alone does not tell us whether our activities doom us, or not. For this, we need better understanding of the causal dynamics of niche construction processes, which evolutionary theory can provide.

A good deal of contemporary work in cultural evolutionary theory focuses on the adaptive significance of culture. This, in turn, leads to what we will call—following Gould & Lewontin [8]—a *Panglossian tendency* in some views of human adaptability*.* Cultural evolution theorists rightly point to the role that human cultural capacities can play in driving human adaptation, making us smarter, more cooperative, and, in some contexts, particularly prosperous [9–11]. A popular trope in this literature is to point to the many ways that human cultural groups have adapted to seemingly extreme environments, such as deserts or the arctic, accompanied by tales of how outsiders lacking the relevant cultural knowledge suffer and die—a comforting view of culture as, literally, a life saver. Indeed, cultural evolution theorists have proposed that climate change itself was one of the primary drivers of the evolution of uniquely human abilities of cultural learning and adaptation—making climate change possibly the very *raison d'être* of human adaptability [12,13]. Although cultural evolutionists are well aware of the potential of cultural evolutionary processes leading to maladaptive outcomes—which we discuss in greater detail below—the Panglossian tendency is distinctly visible in some discussions of contemporary climate change adaptation, where it is argued that human cultural adaptability will save us, just as it has in the past [14].^1^

Within this Panglossian perspective, culture is seen to act as a kind of invisible hand that produces evolutionary optimal outcomes for cultural group members although those outcomes were not intended or foreseen by those group members. Applied to the particular case of climate change, the Panglossian perspective suggests laissez-faire polices of climate intervention in which we are better off letting cultural evolution run its course in finding effective solutions to the climate crisis independently of any intentional changes in collective action or political organization.

In what follows, we will offer a critique of the Panglossian tendency in cultural evolution, and we will highlight the power of cultural practices to generate existential risks both within and across cultural groups. Our aim is not to dispute the centrality of natural selection in the evolutionary process, nor to dispute the role of culture in the course of human evolutionary history and within contemporary human lifeways. Instead, our aim is to motivate a nuanced view of cultural evolutionary dynamics—one according to which cultural evolutionary dynamics can at once increase our ability to adapt while at the same time generate a host of undesirable outcomes, from misery to extinction. In this respect, we will argue that nothing in the cultural evolutionary process itself guarantees human wellbeing or a harmonious fit between human communities and their environments. For that, we need something else that goes beyond the confines of evolutionary theory.

We will begin by saying more about niche construction and cultural evolutionary theory. We then explore the upsides and downsides of evolutionary dynamics in a human niche-constructed world, and what we might or might not expect those dynamics to produce. Building on these considerations, we close the paper by drawing some conclusions about the need for policy and intentional interventions in the dynamical cultural systems that have produced the Anthropocene.

## 2. Niche construction and cumulative cultural evolution

Odling-Smee, Laland and Feldman describe niche construction as the set of processes whereby ‘organisms, through their metabolism, their activities, and their choices, define, partly create, and partly destroy their own niches' ([5], p. 641). Here we will not concern ourselves too much with what a niche is, beyond just the environment itself. Our focus is on niche construction as the reciprocal causal interplay between humans, their activities and the global environment—since the ‘niche' being constructed or modified by humans encompasses the entire biosphere.

A central concept for understanding Anthropogenic global climate change is that of *cumulative cultural evolution*. Cultural evolution refers to changes over time in human practices, ideas, customs and beliefs. This is essentially the same as human history, but with a focus on evolutionary dynamics that shape historical trends, including dynamics of adaptation to environments through, for example, the cultural analogue of natural selection acting on human practices and beliefs through their impacts on survival and reproduction [15]. *Cumulative* cultural evolution refers to processes whereby human practices, knowledge and technologies increase in complexity over time [16]. In the case of human niche construction during the Anthropocene, there is a literal interpretation of the ‘accumulation' of human culture and its products, as seen by the increasing piling-up of human-made stuff across the face of the earth [17].

Cultural evolutionary theorists often point to the bright side of human cultural capacities as the ‘secret of our success' [10]. On this view, our species' unprecedented expansion to inhabit virtually the entire globe is due to our remarkable capacities for cultural learning, cultural transmission and innovation that constantly add to the accumulated store of cultural knowledge. Our ability to wield culture in this way is indeed one of the main sources of our remarkable powers of global transformation. It is at the same time the very source of the human-induced predicament in which we find ourselves, making us, perhaps ironically, the victims of our own success as well. Our ‘success' as a global species is the primary cause of human-induced climate change. Moreover, as many critical commentators on the Anthropocene literature have pointed out, this ‘success' has been both asymmetrically produced and asymmetrically experienced by different groups of people across the globe, as we discuss further below [1,18,19].

From the perspective of cultural evolutionary theory, cultural learning and transmission are the primary means by which we adapt to our environments. This occurs through processes of individual variation and learning coupled with social transmission of knowledge and behaviour across individuals and generations. Cumulative culture, which allows each of us to receive huge masses of cultural knowledge accumulated by generations before us, is also a source of continuous change. For example, technologies for agriculture and food resource extraction become ever-more sophisticated over time, supporting larger populations and creating selection for continuous improvement and innovation.

This takes us to cumulative processes of niche construction. All living things modify and construct their environments through their metabolic processes and through their interactions with things that reside outside the spatial boundaries created by those metabolic processes [20]. In this sense, niche construction is extremely widespread in the biological world. But some processes of niche construction are cumulative in the sense that the activities of one generation in a population have lasting, and sometimes dynamically changing, effects on the selective environments inherited by subsequent generations within that population. The mounds built by some species of termites have this cumulative character: the activity of mound building in one generation is inherited by the next generation, which in turn can make changes to the structure of the mound.

Cumulative niche construction has played a central role in human evolutionary history. To take a particular example, technologies for producing more and better-quality food can support population growth. Population growth leads to demands for more food and to a stronger selective environment for both innovations in food technology and ever-increasing resource extraction (deforestation, animal husbandry) in a self-feeding spiral. In this way, cumulative processes of niche construction can generate a situation in which cultural agents come to modify their environments in an ever-accelerating, runaway fashion [21,22]. The ever-accelerating character of central processes of cumulative cultural niche construction is arguably the source of the ‘great acceleration' in population, technology and resource extraction that has been documented by theorists of the Anthropocene [2]. Many indices of this great acceleration, such as global use of fossil fuels, continue to accelerate upward with no sign of a plateau or inflection point in sight.

An extensive body of research has revealed import connections between cultural adaptation and climatic instability. This work has documented that human evolution took place in the context of a changing climate [17,23], that the need to respond to temporal and spatial variation in climates has been among the selective pressures responsible for complex cognition and flexibility in the human behavioural repertoire, and, in particular, that culture evolved in our lineage at least in part because it allowed rapid adaptation within unpredictable climates [13].

Against this backdrop, Panglossian views of cultural evolution can seem appealing. In particular, it can be tempting to suppose that cultural processes will generate effective solutions to problems of climate change without the need for explicit policy interventions or intentional changes in our practices of collective action. Market-based versions of this perspective hold that human ingenuity finds solutions to problems without the need for any top–down orchestration or coercion; individuals, or firms, merely acting in their own self-interest will find solutions because it is in their interest to do so [24]. Similarly, it can be tempting to suppose that cultural evolutionary processes will produce group-beneficial solutions to climate change dilemmas. After all, there exist formal evolutionary models that show that group-beneficial outcomes can, sometimes, be produced by processes of cultural evolution—typically through an added ingredient of ‘cultural group selection', driven by competition between groups [25]. Within such a perspective, culture operates as a kind of ‘invisible hand', to borrow Adam Smith's metaphor [26], which produces evolutionarily optimal outcomes for group members although those outcomes were not necessarily intended or foreseen by those group members.

However, as cultural evolutionary theorists are aware, group-beneficial outcomes are not guaranteed by, or even a likely outcome of, cultural evolutionary processes except in very special circumstances [9]. Indeed, as we describe below, culture can be a source of both *group-specific maladaptation* but also *across-group existential risk*. We will first describe these undesirable features of cultural evolutionary processes. We then argue that these downsides of cultural evolutionary dynamics are important for understanding feasible policies of climate-change remission.

## 3. Culture as a source of existential risk: the back of the invisible hand

Natural selection refers to the processes whereby variants in a population have a particular distribution because of the contribution of those variants to the relative success, or ‘fitness', of the members of the population. It has long been known that these processes need not produce outcomes that are beneficial for all for even most members of the population, and can even produce outcomes, including stable equilibria, that are sub-optimal for everyone. Processes of runaway selection, for example, can lead to the evolution of costly traits, such as weaponry or ornamentation, that benefit some individuals relative to others but that are a net cost to everyone. This can occur between groups or species as well. Biologists have documented cases of antagonistic coevolution between males and females in the same species, and in arms races between predators and prey or hosts and parasites, where members within or across species pay substantial costs because of the inescapable nature of their conflicts [27]. In principle, runaway selection due to within-species competition can even lead to extinction [28].

The evolution of harmful or costly traits can also occur in cultural evolution. Cultural norms and practices can spread and become stabilized in a population even when they impose a net cost on the people who adopt them. Anthropologists have argued that certain forms of ‘costly signal', such as costly signals of religious devotion or group membership, can spread and become stabilized through competitive arms races in which individuals compete to demonstrate in increasingly harder-to-fake ways what their displays signal [29,30]. Revenge cycles are another form of self-perpetuating and arguably harmful cultural practice, which can form self-stabilizing cultural equilibria from which individuals cannot opt out without potentially paying an even higher cost [31,32].^2^ Runaway selection refers to self-feeding evolutionary spirals that can occur either within populations—as in the escalation of costly signals—or between them, as in the case of arms races from which neither side can unilaterally back down without risking annihilation.

Crucially, in all of these cases natural selection is occurring, and technically, some form of adaptation is occurring too. For example, costly signals of religious devotion can be and are regarded by evolutionary theorists as cultural adaptations to particular social circumstances [29,30]. Similarly, attitudes and values that lead to violence in so-called ‘cultures of honour' could be regarded as adaptive if individuals are better off adhering to them than disregarding them in the local cultural context [31,32]. There are several key points about what ‘adaptation’ means in this context that are important for our understanding of adaptation in the context of the Anthropocene.^3^

First, in an evolutionary context, adaptation is a technical concept that is not equivalent to wellbeing, flourishing or happiness. For example, both reactive and premeditated (or proactive) aggression can be adaptive for humans and yet they often result in outcomes that significantly reduce humans' all-things-considered wellbeing. Questions of what is evolutionarily adaptive can come apart from questions of peoples' subjective preferences and experiences, and from questions of objective harm to individuals, groups or the environment. In a cultural context, some people might regard some cases of self-harm or harm to others as pleasurable or virtuous (e.g. revenge killing), and it could even be adaptive in the evolutionary sense (an option that increases chances of survival or avoiding a greater harm). In other cases, cultural dynamics might lock communities into cycles that they might prefer to escape if they could—and/or, the community could be ‘better off’ in some objective sense without the harmful practices.

A second and related point about adaptation in the evolutionary sense is that what is ‘adaptive' is defined locally, in the sense that an organism acting adaptively would not have higher fitness (survival and reproduction) in the local context if it chose to do something else. This means that evolving systems can get stuck or trapped in sub-optimal equilibria that selection alone will not get them out of [21]. Communities stuck in cycles of violence might prefer to escape, but find themselves unable to do so. At larger scales, societies or other cultural systems could be stuck in sub-optimal equilibria. For example, the healthcare system in the USA might be regarded as a sub-optimal configuration of options that most participants would prefer to be otherwise, but individuals and even groups cannot change it without a critical mass of intentional action.^4^

This second point is particularly important in the case of climate change. Cultural practices that perpetuate harmful environmental effects such as excessive emission of greenhouse gases could be a self-reinforcing stable equilibrium in which all actors are doing what is ‘best' for them, or perceived as best, given their set of choices. In such cases, the cost for individuals or cultural groups to unilaterally defect from their current policy of actions can seem to outweigh the benefits associated with any alternative courses of action. Relatedly, cultural learning itself can make cultural groups increasingly unsuited to adaptively respond to environmental variation. This can be because cultural change does not always take place rapidly enough to cope with environmental change—there can be significant temporal lags between environmental change and cultural change [12,36]. But more centrally, strategies of cultural transmission such as conformist learning can cause a cultural group to adopt policies of action that are so unsuited for the changes taking place in their environment that they result in that cultural group going extinct [37].

Situations in which individual agents acting in their own interest produce a group-level harm have come to be called ‘tragedies of the commons' [38–41]. Structurally similar situations arise in the collective behaviour of cultural groups. In such cases, group-level behaviour that is acquired through adaptive strategies of social learning can produce outcomes that are harmful not just for that group but for other groups as well. We refer to these situations of individual and collective behaviour as the ‘back of the invisible hand', because they consist of scenarios in which the pursuit of local individual and cultural group goods produces outcomes that make those individual and cultural groups worse off than they would otherwise be.

## 4. The cultural evolution of the Anthropocene

Anthropogenic climate change is the result of cumulative cultural practices of human niche construction [6,7,21,42]. We here do not want to take a stand on the ongoing debates about exactly when human practices of niche construction began to impact global climate patterns. Instead, our aim in this section is to briefly highlight the ways in which the *character* and the *scale* of these practices of niche construction derive from patterns of cultural evolution gone bad, or what we have called the back of the invisible hand of culture.

As we noted at the outset of our discussion, all living organisms engage in some form of niche construction. However, the character of human practices of niche construction is quite different from those displayed by other living animals. The emergence of human agricultural practices provides a useful example [43]. We clear and modify landscapes and soil in order to plant crops or provide pasture to domesticated animals. This has profound and reciprocally related effects on both the nature of underlying environments and the agents that occupy those constructed environments. Agricultural practices result in changes to the global climate as ecosystems are converted to farmland and through the byproducts that agriculture produces, which are estimated to constitute 15% or more of global anthropogenic greenhouse gas emissions [44]. Another example of a human-specific practice of niche construction is the much-discussed case of fossil fuel use. In each of these cases, it was through cultural means that these practices of environmental exploitation and modification were discovered and perpetuated. And in each case, what we see are not just single causal ‘steps' but a cumulative spiral of changes in which practices of environmental interaction have been preserved and incrementally updated over time.

This takes us to the scale of human practices of niche construction. Agricultural practices changed within cultural communities, often in the direction of increasing the yield and size of agricultural practices, which in turn increased the size of the populations they support. The result is an acceleration in the amount and degree of landscape transformed. Likewise, fossil fuel use has increased at staggering rates since the industrial revolution and particularly within the past century. Annual CO_2_ emissions are currently estimated to be around 35 billion tons per year—up from 6 billion tons per year in 1960 [45].

The rapidly accelerating character and scale at work of these practices of niche construction are a sign of a self-feeding, autocatalytic system—one that does not appear to be self-correcting (though we may face limits as the amounts of remaining land and fossil fuels available to convert approach zero) [2]. Importantly, however, the factors generating this self-feeding niche construction resemble a tragedy of the commons, where no individual, firm or nation has an incentive to scale back consumption or production. Indeed, the incentive structures of capitalism and global competition push in the opposite direction.

Processes of competition occur at different scales, among different entities and for different reasons. Within nation-states, for example, there is competition between firms for economic growth, and to capture percentages of consumer markets—forces that drive niche-constructing processes such as agriculture, resource extraction, manufacturing and energy consumption. There is competition between individuals that can drive similar processes, as is seen in the competition for real estate and housing that drives up prices, feeds endless cycles of renovation, and expands cities to cover larger and larger areas, with the corresponding construction of infrastructure to support the expansion (among the figures originally used to support the idea of a ‘great acceleration' worldwide were data on the accelerating number of McDonald's restaurants around the globe [46]).

Between nation-states, too, there is competition for political power, growth, and the extraction and control of resources. While these processes are often co-extensive with processes of competition between individuals and firms, between-state competition has the added downside that it can prevent state policies, even if well-intentioned, from reaching optimal global solutions. Competition at all of these levels comes with the challenges of unilateral defection that are characteristic of collective action problems, and that produce tragedies of the commons [40]. Even if some states, for example, adopt policies to cap greenhouse gas emissions in an effort to curb global warming, other states might choose not to, and/or to help national industries find workarounds to international treaties to benefit local economic growth.

Troublingly, intentional policies to incentivize good behaviour by firms, nations and individuals can sometimes create perverse incentives that can make the situation worse, as some case studies of resource management have shown [47,48]. Empirically, seemingly well-intentioned policies designed to shift incentives often do not produce the desired outcomes. For example, carbon offset programmes have been shown to produce an excess of credits for projects that do not actually reduce emissions, for a variety of reasons having to do with the very incentive structures created by the policies [49]. A lesson from this is that the dynamics of natural selection in economic markets can indeed produce ‘adaptation', but often not of the kind we want—despite deliberate, scientifically informed efforts at control. The invisible hand is strong here, and it does not push in the direction we would like.

## 5. Lessons for policies of climate change adaptation

What lessons does the foregoing have for effective policies of climate change adaptation? We will close by offering two primary lessons. The first concerns the need for clarity about the outcomes that policies of climate-change adaptation are trying to promote. The second concerns the importance of structural interventions that reflect contingent details of political history and material inequality in providing cross-cultural means of environmental safeguards and fossil-fuel reduction.

First, on the need for clarity about climate-change adaptation concerns. As we noted in §3, adaptation is a technical concept in evolutionary theory. Adaptation in this technical sense pertains to the process whereby the frequency or distribution of traits in a population—be they biological, cultural, or some other kind of trait—change over time in ways that make the members of that population differentially better able to survive and reproduce. But the term ‘adaptation' can also be used to denote other types of relations that hold between individuals in a population and the environments in which those individuals live. For instance, ‘adaptation’ could refer to the extent to which individuals adopt practices of environmental interaction that better satisfy their preferences or desires than alternative practices of environmental interaction. Alternatively, ‘adaptation' could refer to the extent to which individuals in a population adopt practices of environmental interaction that increase their individual and collective wellbeing in either the current generation or in subsequent generations.

These senses of the term ‘adaptation' are not equivalent, and a population could become adapted in one of these senses without thereby becoming adapted in the others. Discussion of climate-change adaptation would benefit from a more careful elucidation of the specific type or types of adaptation that are being sought after and that policies are intending to promote.

We strongly suspect that it is not adaptation in the technical biological sense that is at issue in the search for policies of climate-change adaptation. For one thing, adaptation in the biological sense is a purely *local* or *within-population* process. And yet, we take it that policies of climate-change adaptation are not purely local in this way. For instance, we assume that most policymakers would endorse a firm constraint according to which their policies of climate-change adaptation should be selected so as to minimize the extent to which those policies reduce the survival and reproductive prospects of members of other populations.

Instead, we suspect that many discussions of climate-change adaptation centre on a notion of adaptation that pertains to relations of human (and broader ecological) wellbeing or flourishing. After all, policies of climate-change adaptation are generally not designed merely to promote survival and reproduction but also to promote the ability of the members of populations to function, develop and thrive. In this sense, while climate-change adaptation is partly about the perpetuation of human and other organismic life, it is also, or should be, about the perpetuation of the *quality* and *character* of those lives.

There is a long-standing set of debates concerning the status of interpersonal comparisons of wellbeing and the extent to which such notions of wellbeing can or cannot be measured or otherwise modelled [50]. We will not try to settle such debates here, although we are sympathetic to approaches that model wellbeing in terms of the ability of agents to develop a distinctive set of *capabilities* in the ecological and cultural environments in which those agents are embedded [51–53]. Our central point is the need to recognize the potential conceptual slippage between biological notions of ‘adaptation' and a variety of broader notions of ‘adaptation' and the need for increased theoretical reflection and debate about the features that make lives—human or otherwise—go well. As we have argued, the factors that increase the wellbeing of a living thing or its descendants do not straightforwardly reduce to factors that increase biological or cultural fitness. This is not to deny that evolutionary theory has much to contribute to an understanding of how we might best respond to the climate crisis. But in so doing, evolutionary theorists will need to move beyond the narrow focus on biological adaptation or cultural group selection and consider the dynamics associated with a broader array of organism–environment relations.

We close now with our second lesson for policies of climate-change adaptation. We have argued that human practices of niche construction do not invariably produce outcomes that are adaptive in either the narrow sense of increasing the ability of agents to survive and reproduce or in the broad sense of increasing the character and quality of those agents' lives. In this sense, we have emphasized the *contingency* of evolutionary processes in producing outcomes that are all-things-considered beneficial to the lives of humans and other organisms.

Our argument provides a general theoretical rationale for the need for planned policies of climate-change adaptation. More specifically, our argument highlights the importance of polices of climate-change adaptation that seek to introduce *structural interventions* into current practices of human environmental interaction.

Technological innovations pertaining to the introduction of novel sources of energy and energy storage—or, alternatively, to novel means of CO_2_ reduction—clearly do have important roles to play in global policies of climate-change adaptation [54]. However, our argument in this paper raises an acute challenge to those who would maintain that such technological innovations will arise in the context of open market national economies or as the natural byproduct of human cultural progress more generally (cf. [24]). For as we have already noted, economic competition between and within cultural groups (whether those groups are nations, corporations or groups of other kinds) generates incentive structures that favour already-established practices of energy production and consumption. These pressures from cultural group competition are amplified by the fact that the effects of climate change are inherently unpredictable. This is especially true in light of what have been called *climate-change cascades* [55–57], in which one type of environmental effect not only triggers another type of environmental effect but also triggers changes in human patterns of migration and intergroup relations. These climate-change cascades can be expected to have profound effects on economic markets and the ability of such markets—unstructured by top–down regulations and protections—to generate technological innovations that serve to remit the climate crisis.

The question of which kinds of structural interventions are both politically feasible and environmentally effective is the subject of an enormous amount of current research, including many of the papers in this issue [58–62]. It is well beyond the scope of our discussion to assess the relative strengths and weaknesses of these policy proposals. However, we do want to note the ways in which a focus on the contingency of the effects of culturally driven human niche construction underscores an often-cited political constraint on policies of climate-change adaptation—namely, that cultural groups and historical entities (states, corporations, etc.) that have contributed more to the problem, should contribute more to viable solutions to the problem [18,55,63].

As many critical commentators in the literature on climate change and the Anthropocene have pointed out, human-induced climate change has neither been uniformly caused by the human population across the globe, nor have its negative consequences been uniformly suffered. Anthropogenic climate change has disproportionately been the result of the activities of some cultural lineages rather than others. In particular, cultural lineages in the Global North (and especially in the USA) have contributed significantly more to the problem than those in the Global South. And many of the worst consequences of the global evolution of the Anthropocene have been or will be suffered by nations and people that have been subject to colonial rule and imperialistic practices [17,18]. Resources have long been and continue to be extracted from the poor and marginalized to improve the comfort and welfare of the wealthy and powerful—often on purpose. As the Anthropocene proceeds, resources become depleted, and the livability and economic potentials of different parts of the earth shift, these differential impacts of human-induced niche construction will continue to proliferate. For example, the melting of Arctic sea ice will be an economic boon to some, and a source of misery, death and displacement for others [64]. Here, too, economic and political power seems likely to accelerate rather than dampen the harmful dynamical processes of niche construction during the Anthropocene.

We believe that these historical facts about the cultural evolution of the Anthropocene provide support for those who have called for *climate change reparations* that provide economic support and tractable means of fossil-fuel reduction and environmental safeguards to communities that have been subject to colonial rule and imperialistic practices [18,55,63]. Whatever the fate of this specific policy proposal, we take our argument to show any discussion of policies of climate change adaptation is incomplete without a recognition of the roles of colonialism and imperialism in shaping the current geopolitical landscape [65].

## 6. Conclusion

Culture may well be the secret to humans’ remarkable success in geographical range and distribution. But culture also gives rise to outcomes that generate substantial risks to the character and quality of the lives of humans and other living organisms. The Anthropocene provides a vivid case in point. A recognition of the role of human cumulative cultural evolution—and of human cultural niche construction—in the rise of the Anthropocene leads to a more nuanced understanding of both the prospects and the perils of cultural evolution. More significantly, we have argued that taking seriously the sometimes negative and harmful aspects of cultural evolutionary dynamics is an important addendum to discussions of climate-change adaptation, and is crucial for a realistic understanding of the conditions under which climate-change adaptation is likely to occur. While cultural evolution and adaptation are important concepts for understanding the situation in which we find ourselves with respect to climate change, it may be more fruitful to regard cultural evolutionary dynamics as a set of constantly moving structural constraints within which intentional efforts at change must be enacted.

## Data Availability

This article has no additional data.

## Endnotes

^1^ For example, consider the following passage from Lomborg [14, p.159], which strikes us as Panglossian to the point of absurdity: ‘This is why we need to adapt to a warmer planet over the coming decades. Fortunately, humanity has remarkable adaptive capacity. There are people living in the icy extremes of Siberia and northern Canada, in the burning hot Sahel desert and Australian Outback, in the dry Atacama desert plateau of South America, and in the rain-soaked Meghalaya state in India. Not only do people withstand significant variations in temperature and rainfall, but falling per capita death rates from natural disasters show that we have more resilience today than ever before. At its simplest, adaptation means that people react sensibly to challenges—in our case, to a changing climate. As it gets warmer, more people will adapt by turning on their air conditioners (and fewer will use their heaters). If they don't yet have an air conditioner, more people will buy one (and more people will be able to buy one as global prosperity increases). Similarly, tourists will adapt to a warming world by changing their travel destinations. Warm places like Sri Lanka will host fewer tourists. On the other hand, more visitors will choose Finland and Canada for their next holiday, while fewer Finns and Canadians will travel abroad’.
^2^ We note, however, that the question of whether cultural practices are harmful and to whom can be complex and to answer, and perceptions of those inside or outside the community might not be sufficient to answer it [33].
^3^ Jones *et al.* [34] discuss the ‘negative valence’ of adaptation associated with the view that adaptation is a secondary line of defense once efforts at mitigation have failed. Here we focus on an even more negative aspect of the concept of adaptation: adaptation, in the biological sense, can be terrible for individuals, groups and everyone else. We attempt to explain what we mean by this perverse, but important, implication of adaptation by natural selection.
^4^ Wilson *et al.* [35] distinguish between ‘Type 1’ complex adaptive systems (CAS1), which are ‘adaptive as systems’, (e.g. the immune system), and ‘Type 2’ complex adaptive systems (CAS2) that are ‘composed of agents following their respective adaptive strategies’ (they cite ecosystems as an example). They point out that ‘there is little awareness that CAS2 systems can be profoundly maladaptive as whole systems’, echoing our point here.
